# Core training and performance: a systematic review with meta-analysis

**DOI:** 10.5114/biolsport.2023.123319

**Published:** 2023-02-03

**Authors:** Ángela Rodríguez-Perea, Waleska Reyes-Ferrada, Daniel Jerez-Mayorga, Luis Chirosa Ríos, Roland Van den Tillar, Ignacio Chirosa Ríos, Dario Martínez-García

**Affiliations:** 1 Department of Physical Education and Sport, Faculty of Sports Sciences, University of Granada, Granada, Spain; 2 Exercise and Rehabilitation Sciences Laboratory, School of Physical Therapy, Faculty of Rehabilitation Sciences, Universidad Andres Bello, Santiago 7591538, Chile; 3 Department of Sports Science and Physical Education, Nord University, Levanger, Norway

**Keywords:** Trunk, Jump, Throw, Velocity, Balance, Core stability

## Abstract

The purposes were to synthesize as much scientific evidence as possible to determine the effect of core training on balance, throwing/hitting velocity or distance, and jumping in healthy subjects, identify the possible differences between isolated and combined core training on performance and study training and sample variables related to performance. PRISMA guidelines were followed, and a systematic search was performed in the Scopus, Web of Science, Sports Discuss, and PubMed databases with no date restrictions until November 2022. The studies were considered for this meta-analysis following PICO; a) randomized control trials and randomized allocation studies with healthy subjects and > 12 years old b)isolated or combined core training programs with a minimum of 4 weeks in length; c) athletic performance outcomes for balance, throw/hit, and jump variables should be measured; d) sufficient data to calculate effect sizes. The Cochrane Collaboration Risk of Bias Tool and the Grading of Recommendations Assessment, Development, and Evaluation approach were used for assessing methodological quality. A total of 3223 studies were identified, 22 studies were included in the systematic review and 21 for the meta-analysis. We observed that core training improved balance outcomes (ES = 1.17; p < 0.0001), throwing/hitting velocity (ES = 0.30; p = 0.14), throwing/hitting distance (ES = 3.42; p = 0.03), vertical jumping (ES = 0.69; p = 0.0003), and horizontal jump (ES = 0.84; p = 0.01). Our findings indicate that core training improved different variables of performance such as balance, throw/hit, and vertical and horizontal jump.

## INTRODUCTION

The core represents the functional term of the trunk muscles, encompasses the back, abdominal, pelvic floor, diaphragm, hip, and gluteus muscles, and connects the upper and lower extremities. These muscles are responsible for providing stability to the spine and transferring forces from the proximal area to the most distal area of the body [1, 2]. Core research has focused on the study of core stability in injury prevention [3]. Several studies analyze the relationship between stability and core strength with anterior cruciate ligament injury [4], low back pain [5], and hip trochanteritis [6].

Furthermore, it explored how core training affects sports performance [7]. During the last decades, the focus of core study has been placed on performance and the influence of this musculature in actions such as balance, jumping, hitting or throwing, jumping, and running. Granacher et al. observed correlations between trunk muscle strength and balance variables and considered that core strength training is a feasible training program for seniors [8]. Prieske et al. concluded that core training has a small effect on balance and a medium effect on muscle power. Reed et al. concluded that performance in sports movements like a golf swing or running improves with core training and that there is little correlation between core stabilization and performance [9, 10]. However, this review did not focus on any specific indicator of sports performance; instead, the authors examined the effects on general performance, lower-extremity performance, and upper-extremity performance [9].

In addition, core training improves proximal stabilization, essential in sports requiring a high velocity of the distal segment, as in golf, tennis, baseball, handball, football, etc. [11–13]. In these sports, movement patterns are based on sequential kinetic chains and pursue a high final velocity of the distal segment. Via the kinetic chain, angular momentum is transferred from one segment to the other; thus, correct positioning of the pelvis and flexion and torsion movements of the trunk have confirmed the beneficial effects in baseball, handball, volleyball, and tennis [14–20].

A recent meta-analysis examines the effects of trunk muscle training on physical fitness and sport-specific performance in healthy competitive athletes. They found moderate effects on trunk muscle endurance, linear sprint speed, and change of direction or agility, a large effect on local muscle endurance, small effects on maximal muscle strength, and, finally, moderate effects on sport-specific performance [21]. In addition, lower body muscle power was analyzed including the variable jump, and a small effect in favor of core training was found. However, this study did not take into account the type of jump, horizontal or vertical, to analyze the effect of core training on performance variables.

Even though numerous studies have shown the benefits of core training, whether performed in isolation or combined with lower-extremity exercises, upper-extremity exercises, or both [22–24], the impacts of core training on its own on an individual’s performance have not yet been well established. There is great interest in knowing how the core strength acts in the improvement of different performance variables. However, at the moment, an answer to this question has not been obtained. Therefore, this meta-analysis aims to synthesize as much scientific evidence as possible to determine the effect of core training on balance, throwing/hitting velocity or distance and jumping in healthy subjects and identify the possible differences between isolated core training (ICT) and combined core training (CCT) on performance and study intervention and sample variables related to performance.

## MATERIALS AND METHODS

**FIG. 1:**
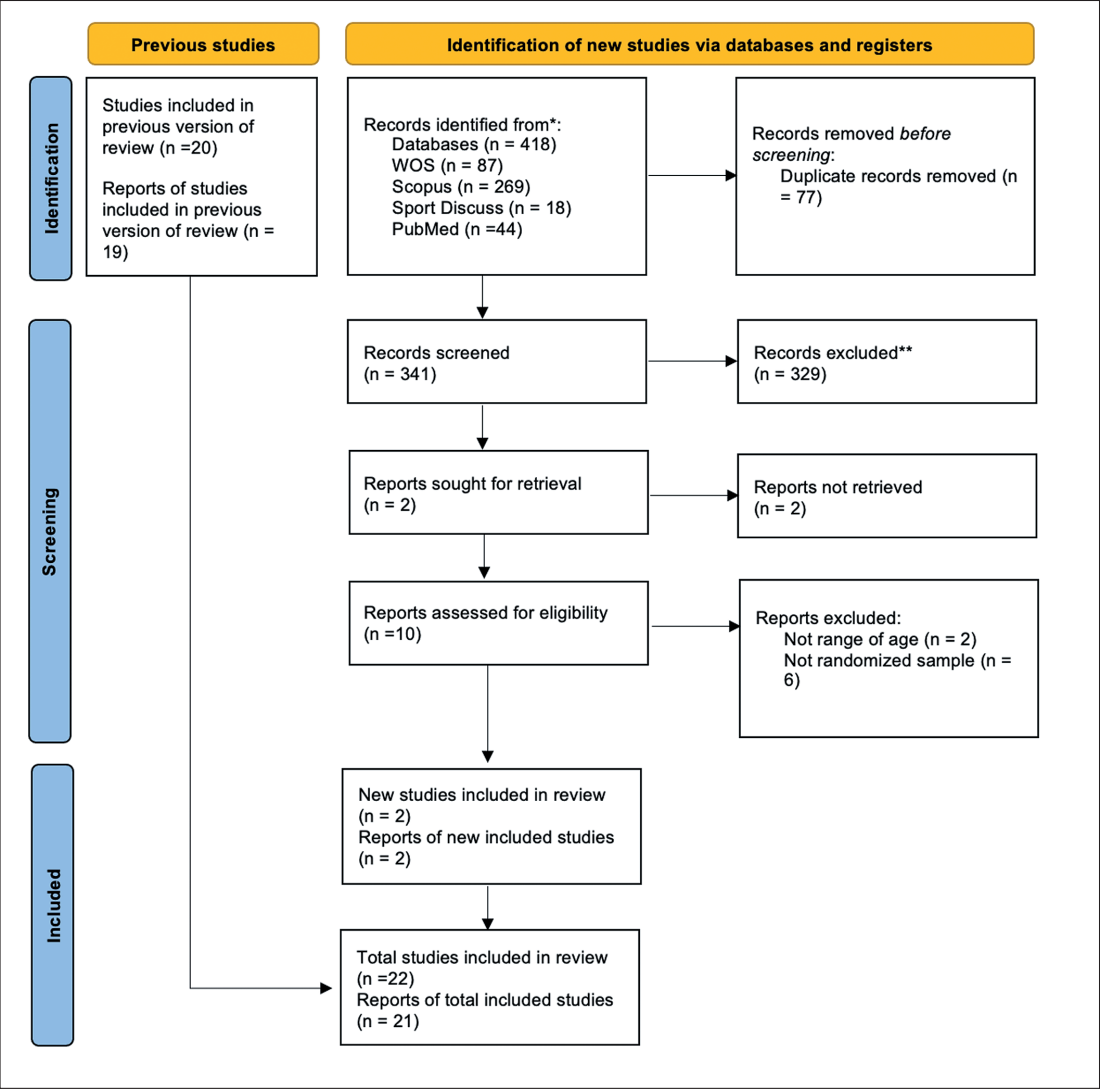
PRISMA flow diagram for updated systematic reviews.

A systematic revision was carried out on 7^th^ September 2021 and updated on 22^nd^ November 2022 to search the published scientific evidence and understand how the core training programs affect athletic performance. The protocol for this systematic review was registered on PROSPERO (Registration No: CRD42021281007). The reporting flow diagram of this systematic review was based on the Preferred Reporting Items for Systematic reviews and Meta-Analyses (PRISMA) guidelines [25] (Figure 1).

### Search Strategy

Randomized control trials and randomized allocation studies were identified by searching the four electronic databases: Web of Science, SCOPUS, SportDiscuss, and PubMed. Before the bibliographic search, a scoping search was carried out to identify possible keywords. Identifying both Medical Subject Headings (MeSH) terms and the following keywords: “core muscle”, trunk, “torso”, abdominal, “endurance training,” stability, “resistance training,” “strength training”, “plyometric exercise”, “athletic performance”, performance, and “physical test”. These search terms were combined with two Boolean operators AND, and OR (Supplementary Table 1). The complete search phrase with the Boolean operators was ((“core muscle” OR “torso” OR trunk OR abdominal) AND (“endurance training” OR stability OR “resistance training” OR “strength training” OR “plyometric exercise”) AND (“athletic performance” OR “physical test”)) to identify English or Spanish language original research studies. Also, the bibliographies of other previous related reviews and the selected studies were examined to search for new studies. Other possible scientific evidence related to the subject was identified by emailing the authors of the published articles.

Two independent reviewers (AR-P and WR-F) examined the title/summary of the articles found in the databases. After the initial selection, they analyzed each study with the inclusion criteria. Each criterion was evaluated as yes/no. In case of disagreement among researchers, the consensus approach was used. The authors were familiar with the existing literature and did not have a different bias toward any of the studies selected for inclusion in the review. A new search (n = 418) was performed on November 2022 to find relevant articles and two articles met the inclusion criteria.

### Inclusion Criteria

Articles that met the following criteria were included in this review: a) only randomized control trials and randomized allocation studies with healthy subjects > 12 years old were considered for this meta-analysis; b) isolated or combined core training programs with a minimum of four weeks in length; c) athletic performance outcomes for balance, throw/hit, or jump variables should be measured; d) sufficient data to calculate effect sizes (ESs). The articles that met the inclusion criteria were identified, and their full-text versions were obtained.

Articles with one or more of these criteria were excluded: a) legal or illegal ergogenic aids or supplementation were used during interventions; and b) cohort or narrative reviews.

### Data Collection Process

All calculations were conducted using Microsoft Excel (Microsoft, Redmond, WA, USA) spreadsheet containing data extracted from each publication. For studies that only reported their data in charts, WebPlotDigitizer version 4.4 was used to obtain the athletic performance values [26]. Review Manager (RevMan) version 5.4.5 was used for all statistical analyses of forest plots. The Cochran Q statistic [27] was used to assess heterogeneity between studies. Heterogeneity is a measure of the differences in main effects between studies. Also, I^2^ statistics were used to evaluate heterogeneity (*I*^2^ > 50%).

Core training program effects on athletic performance were calculated for each included study following both groups’ coding of post-interventions means and standard deviations (SDs). The standardized mean difference (SMD) was calculated by obtaining the post-intervention means and standard deviations of athletic performance values. Studies were grouped into different subgroups according to moderating variables to determine the effect of these variables on the overall effect size (type of core training, intervention-related variables, and sample-related variables). Data were required to take these forms: a) mean and SDs (pre-and post-intervention); b) 95% confidence interval (CI) data for post-intervention change for each group; or when this was unavailable, c) actual p values for post-intervention change for each group; or if only the level of statistical significance was available, d) default p values (e.g., p < 0.05 becomes p < 0.049, p < 0.01 becomes p < 0.0099, and p when not significant becomes p > 0.05). A random-effects inverse variance (IV) was used to calculate de Standardized Mean Difference (SMD).

The analysis of ES was conducted with a random-effects model estimated using the DerSimonian and Laird method [28]; a random-effects model is incorporated when the assumption is that the effect across studies is randomly situated about a central value. Forest plots were generated to demonstrate the study-specific post-training performance differences and ESs within the respective 95% CIs. Combining estimates then allowed for the assessment of a pooled effect. The reciprocal sums of two variances were accounted for, including the estimated variance associated with the study and the estimated variance component due to the variation between studies. Risk of Bias (RoB) and Grading of Recommendations Assessment, Development and Evaluation approach (GRADE) analysis were conducted to identify the presence of highly influential studies that might have biased the analysis.

The study-specific weight was derived as the inverse of the square of the respective standard errors. The ESs of ≤ 0.2, ≤ 0.5, ≤ 0.8, and ≥ 0.8 were considered trivial, small, moderate, and large, respectively [29].

## RESULTS

**FIG. S1:**
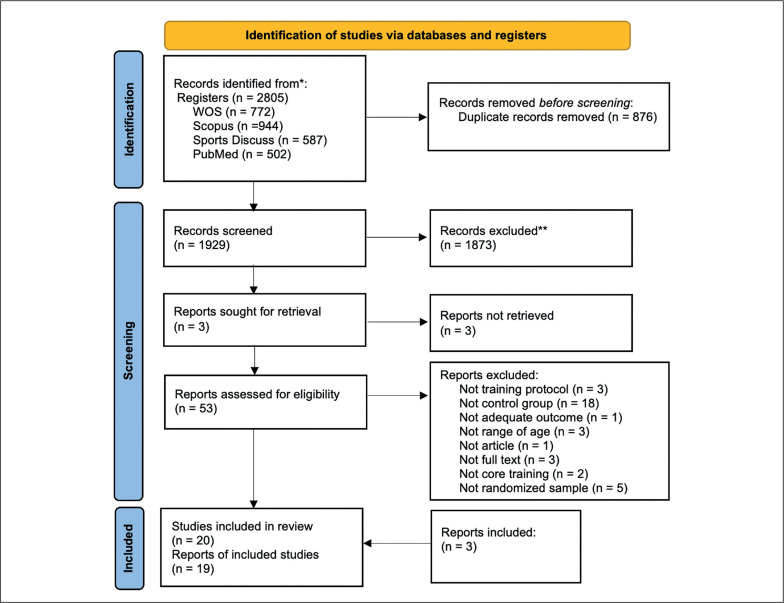
PRISMA flow diagram for new systematic reviews.

The flow diagram of this article’s search and selection is depicted in Figure 1 with the updated search and the original search (Supplementary Figure 1), from “potentially relevant” to “finally included.”

### Study Selection

The preliminary search yielded 1929 relevant abstracts and citations. The full text of 53 articles was deemed to meet the inclusion criteria. Of these, 36 studies were rejected for this meta-analysis due to the reasons seen in figure 1, and three studies were included through manual search. Finally, a total of 20 studies met the inclusion criteria [30–49] for one or all athletic performance variables for the revision, and 19 studies met the inclusion criteria for meta-analysis. One article was not included in the meta-analysis because the sample size in the experimental and control groups was not specified [50]. The updated search was conducted in November 2022 and 418 articles were found with only 2 meetings the inclusion criteria [51, 52].

### Demographic Characteristics

The studies included 172 participants in balance studies (51.74% male, 30.81% female, and 17.45% not reported), 280 participants in velocities or distance throwing/hitting studies (87.86% male and 12.14% female), and 483 participants in jump studies (54.24% male and 45.76% female). The study that did not report the data was the same study that could not be included in the meta-analysis because it did not indicate the sample size in the control and experimental groups. The age range varied between studies, ten studies evaluated youth (13 ≤ 18 years), and twelve studies evaluated adults (18–65 years). The sample comprises students and athletes who practice different sports such as volleyball, baseball, basketball, golf, tennis, handball, soccer, karate, and gymnastic, and the competitive level ranges from recreational to professional (Table 1).

**TABLE 1:** Demographics characteristic of included studies

Authors	Sample	Sex	Age (years)	Sports	Performance variable
Anant et al., 2020	N = 55 EG = 30 CG = 25	M	EG = 25.3 ± 1.52 CG = 26.4 ± 1.63	Volleyball, basketball, kabaddi, handball, kho-kho and football	Standing Broad Jump Test
Arslan et al., 2021	N = 38 EG = 20 CG = 18	M	EG = 16.30 ± 0.47 CG = 16.5 ± 0.59	Soccer	CMJ, SJ and Y-Balance Test
Bashir et al., 2019	N = 30	-	EG = 15.20 ± 0.41 CG = 15.53 ± 1.06	Tennis	SEBT
Benis et al., 2016	N = 28 EG = 14 CG = 14	F	EG = 20 ± 2 CG = 20 ± 1	Basketball	Y-Balance Test
Dello Iacono et al., 2014	N = 20 EG = 10 CG = 10	M	EG = 18.7 ± 0.67 CG = 19 ± 0.63	Soccer	SEBT
Fadhil et al., 2013	N = 25 EG = 13 CG = 12	M	13.28 ± 0.45	Soccer	SLJ and sargent jump test
Fernandez-Fernandez et al., 2013	N = 30 EG = 15 CG = 15	M	EG = 13.2 ± 0.6 CG = 13.2 ± 0.5	Tennis	Serve velocity
Filipa et al., 2010	N = 16 EG = 9 CG = 7	F	EG = 15.4 ± 1.5 CG = 14.7 ± 0.8	Soccer	SEBT
Kabadayi et al., 2022	N = 29 EG = 16 CG = 13	M-F	EG = 12.75 ± 0.77 CG = 13.00 ± 0.91	Karate	CMJ and SLJ
Karagianni et al., 2020	N = 23 EG = 12 CG = 11	F	EG = 13.2 ± 1.3 CG = 12.3 ± 1.3	Gymnastic	CMJ and DJ
Kuhn et al., 2019	N = 20 EG = 10 CG = 10	F	EG = 24.1 ± 3.8 CG = 23.7 ± 5.2	Handball	Throwing velocity
Lu et al., 2022	N = 183 EG = 92 CG = 91	M-F	EG = 14.59 ± 1.00 CG = 14.46 ± 1.78	Students	Long jump
Manchado et al., 2017	N = 30 EG = 15 CG = 15	M	EG = 18.5 ± 3.0 CG = 18.9 ± 3.8	Handball	Throwing velocity
McCurdy et al., 2014	N = 35 EG = 17 CG = 18	M-F	23.43 ± 5.2 (F) and 27.95 ± 7.5 (M)	Tennis	Serve velocity
Mills et al., 2005	N = 20 EG = 10 CG = 10	F	EG = 20.3 ± 2.0 CG = 19.4 ± 1.7	Basketball and voleyball	Sargent jump test
Mills et al., 2005	N = 20 EG = 10 CG = 10	F	EG = 18.9 ± 1.1 CG = 19.4 ± 1.7	Basketball and voleyball	Sargent jump test
Ozmen et al., 2020	N = 20 EG = 10 CG = 10	M	EG = 14.90 ± 0.31 CG = 14.90 ± 0.56	Handball	Throwing velocity, SEBT and SJ
Sato et al., 2009	N = 20 EG = 12 CG = 8	M-F	EG = 37.75 ± 10.63 CG = 39.25 ± 10.81	-	SEBT
Sharma et al., 2012	N = 40 EG = 20 CG = 20	M-F	EG = 21.8 ± 1.8 CG = 22.4 ± 1.8	Voleyball	CMJ and SJ
Sung et al., 2016	N = 40 EG = 20 CG = 20	M	EG = 23.05 ± 0.5 CG = 24.0 ± 1.0	Golfers	Drive distance
Sung et al., 2016	N = 40 EG = 20 CG = 20	M	EG = 23.2 ± 0.6 CG = 24.0 ± 1.0	Golfers	Drive distance
Szymanski et al., 2007	N = 49 EG = 25 CG = 24	M	EG = 15.3 ± 1.2 CG = 15.4 ± 1.1	Baseball	Bat swing and medicine ball hitter’s throw
Taskin 2016	N = 40 EG = 20 CG = 20	F	EG = 19.05 ± 1.15 CG = 18.55 ± 0.76	Soccer	Vertical jump and SLJ
Weston et al., 2013	N = 36 EG = 18 CG = 18	M	47 ± 12	Golfers	Club-head speed

EG = experimental group; CG = control group; M = male; F = female; CMJ = countermovement jump; SJ = squat jump; SLJ = standing long jump; SEBT = Star Excursion Balance Test; DJ = drop jump

In balance performance, females and males obtained a large effect (ES = 1.99; ES = 0.98) respectively in favor of core training. In throwing/hitting performance at velocity or distance males had a large effect (ES = 1.42), however, in females only one study analyzed the effect of core training, obtaining a small effect (ES = 0.24) in favor of training. And for jumping performance, females and males obtained a large effect (ES = 0.96; ES = 1.13) respectively in favor of core training. Furthermore, age affected performance, with adults having a large effect on all performance variables (ES > 0.80) except to jump performance ( ES = 0.67) and youth having a large effect on balance (ES = 1.00), a small effect on throwing/hitting performance at velocity or distance (ES = 0.35) and a moderate effect on jumping (ES = 0.79) with non-significant differences (p > 0.05) (Table 4).

### Measures

The three performance variables (balance, throwing/hitting, and jumping) were evaluated with different tests and instruments (Table 1). The most widely used device for measuring velocity or distance throwing/hitting was radar [30, 37, 39, 40, 46]. Three studies used other measurement devices: a motion capture system [15], a portable launch monitor [42], and a photocell array system [44]. The Star Excursion Balance Test (SEBT) was the most used on balance performance, and two studies used the de Y-Balance test [34, 48]. Jump performance was divided into a vertical jump and a horizontal jump, and there were various tests to measure this variable. The most used vertical jump was Counter Movement Jump (CMJ), Squat Jump (SJ), and Sargent Jump test, and to horizontal jump was the Standing Long Jump test (SLJ). The measuring instrument most used was a contact mat [35, 40, 41]. Other instruments used were an OptoJump System [47], a portable force plate [48], takei-brand jump meter [51], and a meter [36].

### Training

The training intervention was core strength or core stability exercises, and the control group continued with the standard sports training in most of the studies. In some studies, ICT has been performed [31, 33, 35–38, 40, 42–45], and in others, CCT with plyometric training or lower or upper extremity training [30, 32, 34, 39, 41, 42, 46–48]. The most used implements for this type of training are the swiss ball or medicine ball. The duration of the programs ranged between 4 and 12 weeks, being the most frequent program duration of 6–8 weeks with 2–3 sessions per week. Regarding the training sessions, the time of each session was only specified in 13 articles; in these, it ranged from seven to 60 minutes. Finally, 14 studies were carried out in a period of competition or a rest period (off-season or pre-season), while this timing was not specified in the other cases (Table 2).

**TABLE 2:** Details of core intervention protocols of included studies.

Study		Training						Core test
Authors	Design	Type of intervention	Implements	Duration	Time (min)	Frecuency(days/week)	Period of training	Measure of core strength
Anant et al., 2020	RAN	Core muscle strength training	Swiss ball	8 weeks	10–15	5	-	Lateral trunk endurance Curl-up test
Arslan et al., 2021	RAN	Core strength training + small sized games	Without implements	6 weeks	-	3	Off-season	No
Bashir et al., 2019	RAN	Core training program	Swiss ball	5 weeks	-	3	-	No
Benis et al., 2016	RAN	Core stability and plyometrics	Without implements	8 weeks	30	2	-	No
Dello Iacono et al., 2014	RAN	Core stability	Unstable planes	4 weeks	-	5	Season	No
Fadhil et al., 2013	RAN	Core stability, balance, plyometrics and strength	Without implements	12 weeks	20–25	5	Season	Prone Hold Test
Fernandez-Fernandez et al., 2013	RAN	Core training exercises, elastic tubing and medicine ball	Elastic tubing and medicine ball	6 weeks	50	3	Season	No
Filipa et al., 2010	RAN	Core stability and lower extremity	Swiss ball, bosu and airex pads	8 weeks	45	2	Season	No
Kabadayi et al., 2022	RCT	Core strength training program and Sport-specific program	Without implements	8 weeks	30–35	3	Off-season	Flexor, back and lateral endurance test
Karagianni et al., 2020	RAN	Strength training (core + upper limb + lower limbs) and plyometrics	Kettlebell, medicine ball and resistance band	10 weeks	7–9	3	Pre-season	No
Kuhn et al., 2019	RCT	Core stability	Swiss ball	6 weeks	45	2	Season	Swiss Olympic Medical center core performance test battery.
Lu etl al., 2022	RCT	Core strength training + bobby jump	Without implements	12 weeks	7–10	3	-	Core endurance
Manchado et al., 2017	RAN	Strengthening lumbopelvic region	Swiss ball	10 weeks	10–25	3	Season	No
McCurdy et al., 2014	RAN	Core strength and muscular endurance	Medicine ball	8 weeks	15	2	Season	Plank assessment
Mills et al., 2005	RCT	Lumbopelvic stability	Without implements	10 weeks	-	4	-	Lumbopelvic stability
Ozmen et al., 2020	RAN	Core strengthening exercise	Physioball and medicine ball	6 weeks	-	2	Season	No
Sato et al., 2009	RAN	Core strength training	Stability ball	6 weeks	-	4	Season	No
Sharma et al., 2012	RAN	Core strengthening exercise	Without implements	9 weeks	30–40	5	-	Modified double straight leg lowering test
Sung et al., 2016	RAN	Combined strengthening and core exercise	Dumbeell	8 weeks	60	6	Season	Flexion and extension trunk isokinetic strength
Szymanski et al., 2007	RAN	Rotational and full body medicine ball exercise	Medicine ball	12 weeks	-	3	Off-season	Torso rotational strength with isokinetic machine
Taskin 2016	RAN	core training program	Without implements	8 weeks	-	3	-	No
Weston et al., 2013	RAN	Isolated core training	Without implements	8 weeks	-	3	-	Isometric flexor endurance

RAN = randomized allocation; RCT = randomized controlled trial; min = minutes.

### Risk of Bias

**FIG. 2:**
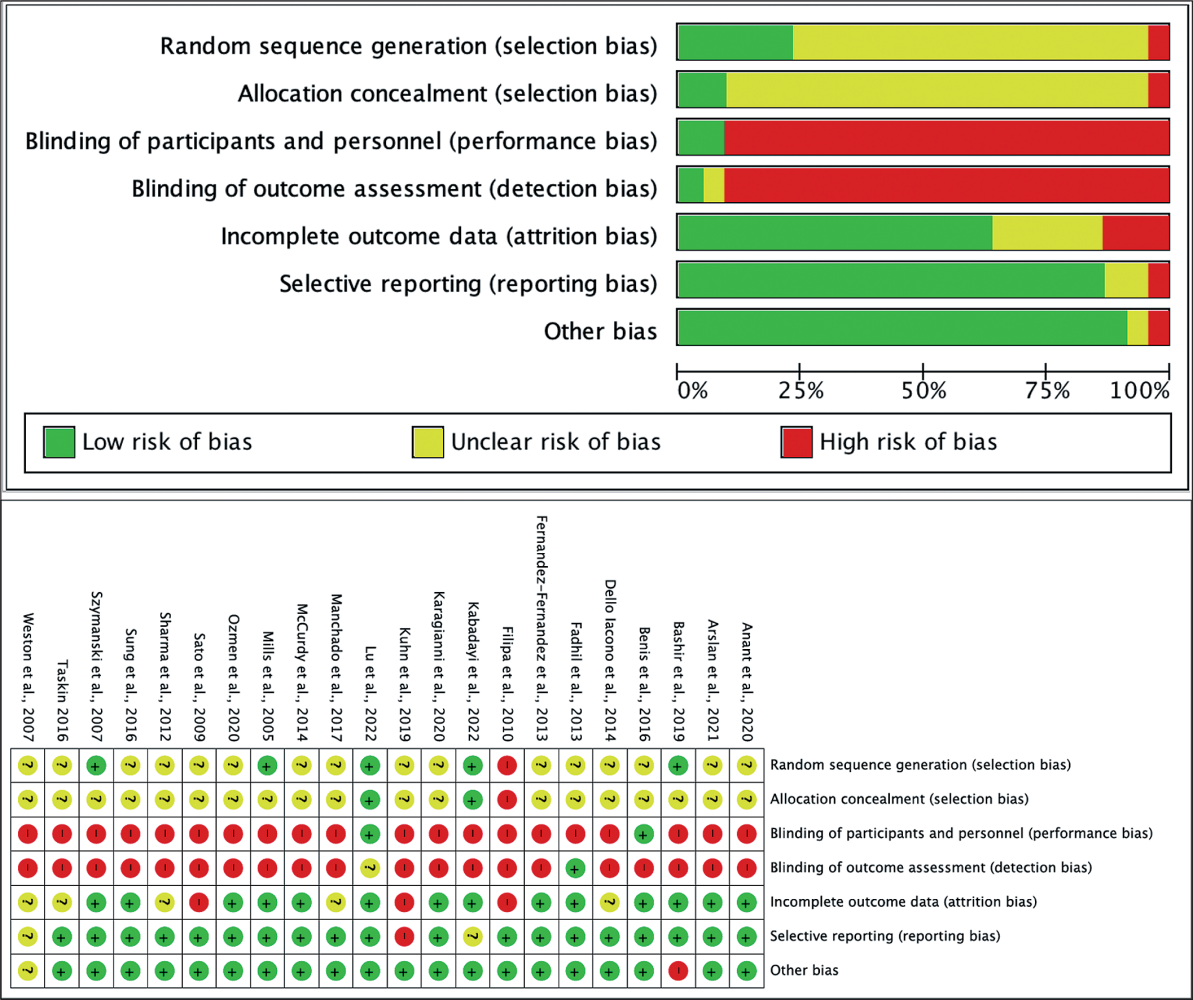
Risk of bias graph and summary.

The risk of bias (RoB) assessment was performed using the Cochrane RoB tool [53]. This tool assesses the RoB according to the following seven domains: random sequence generation, allocation concealment, blinding of participants and personnel, blinding of outcome assessment, incomplete outcome data, selective reporting, and other sources of bias (Figure 2). Each domain could be considered as “low,” “unclear,” or “high” RoB. Data extraction and quality assessment were independently performed by two reviewers (AR-P and WR-F). In addition, a third researcher was consulted for the case in which a consensus could not be reached (DM-G).

### Rating the quality of evidence

The quality of the evidence was rated using the GRADE approach [54]. GRADE offers four levels of evidence: High, moderate, low, and very low. The GRADE pro system (https://www.gradepro.org) was used for each outcome from the meta-analysis to create a summary of the findings table.

### Effects of Core Training Programs on Balance Performance

**FIG. 3:**
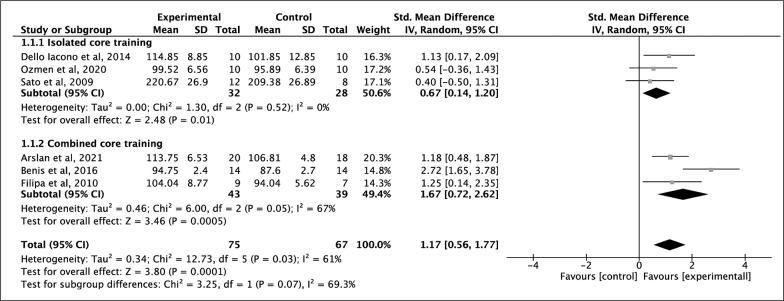
Forest plot of comparison of balance for isolated core training and combined core training vs control group.

Between groups, performance differences were assessed via meta-analysis for six included studies. All six studies showed a positive effect of core training on balance and moderate quality of evidence according to the GRADE rating (Table 3). The SMD 1.17 points (p < 0.001; *I*^2^ = 61%) indicated large effects in favor of core training with a significant difference (Figure 3). The pooled mean ES estimated balance comprised six treatment groups from 6 studies [31–34, 40, 48] (Table 1).

**TABLE 3:** Summary of findings (SoF) and quality of evidence (GRADE) for core training.

Certainty assessment							№ of patients		Effect		Certainty	Importance
№ of studies	Study design	Risk of bias	Inconsistency	Indirectness	Imprecision	Other considerations	core training	CG	Relative (95% CI)	Absolute (95% CI)		
Balance												
6	randomised trials	not serious	serious^a^	not serious	not serious	none	75	67	-	SMD 1.17 SD higher (0.56 higher to 1.77 higher)	⨁⨁⨁◯ Moderate	IMPORTANT
Vertical Jump												
11	randomised trials	not serious	serious^b^	not serious	not serious	none	171	162	-	SMD 0.69 SD higher (0.32 higher to 1.06 higher)	⨁⨁⨁◯ Moderate	IMPORTANT
Horizontal jump												
5	randomised trials	not serious	very serious^c^	not serious	not serious	none	171	161	-	SMD 0.84 SD higher (0.16 higher to 1.52 higher)	⨁⨁◯◯ Low	IMPORTANT
Velocity throw												
7	randomised trials	not serious	serious^d^	not serious	serious^e^	none	110	110	-	SMD 0.3 SD higher (0.1 lower to 0.71 higher)	⨁⨁◯◯ Low	IMPORTANT
Distance throw												
3	randomised trials	not serious	very serious^f^	not serious	not serious^g^	none	65	64	-	SMD 3.42 SD higher (0.35 higher to 6.49 higher)	⨁⨁◯◯ Low	IMPORTANT

CI: confidence interval; SMD: standardised mean difference. Explanations:

a . The heterogeneity between studies was substantial (I^2^ = 61%);

b . The heterogeneity between studies was substantial (I^2^ = 62%);

c . The heterogeneity between studies was considerable (I^2^ = 85%);

d . The heterogeneity between studies was substantial (I^2^ = 53%);

e . Requires a total sample size of approximately 220;

f . The heterogeneity between studies was considerable (I^2^ = 97%);

g . The confidence interval are large (0.35–6.49)

**TABLE 4:** Effects of intervention-related variables and sample-related variables on performance.

	Balance			Throwing/hitting velocity or distance			Jumping		

Intervention variables	S	ES (p-value)	*I*^2^(%)	S (EG)	ES (p-value)	*I*^2^(%)	S (EG)	ES (p-value)	*I*^2^(%)
**Frecuency**									

≤ 2 d/w	3	1.48 (0.02)	79	3	0.23 (0.32)	0	1	0.23 (0.61)	NA
> 2 d/w	3	0.95 (0.0001)	0	5(7)	1.55 (0.007)	94	9(15)	0.98 (<0.0001)	82
		p = 0.45			p = 0.03				

**Duration**									
≤ 6 weeks	4	0.86 (0.0001)	0	3	0.61 (0.01)	0	2(3)	0.90 (0.001)	37
> 6 weeks	2	1.99 (0.007)	72	5(7)	1.41 (0.01)	94	8(13)	0.71 (0.0002)	74
		p = 0.14			p = 0.20			p = 0.57	

**Implements**									
With implements	4	0.78 (0.001)	0	7(9)	1.21 (0.009)	92	3	0.81 (0.0005)	12
Without implements	2	1.89 (0.01)	82	1	0.45 (0.05)	NA	7(13)	0.74 (0.0001)	76
		p = 0.17						p = 0.81	

**Session duration**									
≤ 30 minutes	1	2.72 (< 0.0001)	NA	2	0.24 (0.34)	0	4(5)	1.05 (0.002)	81
> 30 minutes	1	1.25 (0.03)	NA	3(4)	2.68 (0.02)	95	2(4)	0.08 (0.65)	0
					p = 0.03			p = 0.01	

**Total volume**									
≤ 16 sessions	3	1.48 (0.02)	79	3	0.23 (0.32)	0	1	0.23 (0.61)	NA
> 16 sessions	3	0.95 (0.0001)	0	5(7)	1.55 (0.007)	94	9(15)	0.77 (< 0.0001)	73
**Sample**	p = 0.45				p = 0.03				

**Sex**									
Female	2	1.99(0.007)	72	1	0.24 (0.59)	NA	3(5)	0.96 (0.001)	61
Male	3	0.98(0.0001)	0	5(7)	1.42 (0.005)	93	4(6)	1.13 (< 0.0001)	35
		p = 0.19						p = 0.63	
Mixed	1	0.40 (0.38)	NA	1	0.07 (0.85)	NA	3(5)	0.72 (0.10)	90

**Age**									
Youth (12 ≤ 18 years)	3	1.00(0.0001)	0	3(4)	0.35 (0.32)	75	6(9)	0.79 (0.0003)	71
Adults (18–65 years )	3	1.39 (0.04)	81	5(6)	1.74 (0.01)	94	4(7)	0.67 (0.01)	75
		p = 0.58			p = 0.07			p = 0.73	

ES = effect size; S (EG) = number of studies (EG = experimental groups); NA = not applicable; d/w = days per week;

Six studies, comprising two intervention groups, were included in the analyses to determine the effects of ICT or CCT on balance. The weighted mean SMD of 0.67 (p = 0.01; *I*^2^ = 0%) indicated a moderate and significant effect in favor of ICT and the weighted mean SMD of 1.67 (p < 0.0001; *I*^2^ = 61%) indicated a large and significant effect in favor of CCT (Figure 3). In terms of intervention-related variables, no significant differences were found between studies (p > 0.05) (Table 4).

### Effects of Core Training Programs on Throwing/Hitting Performance

**FIG. 4:**
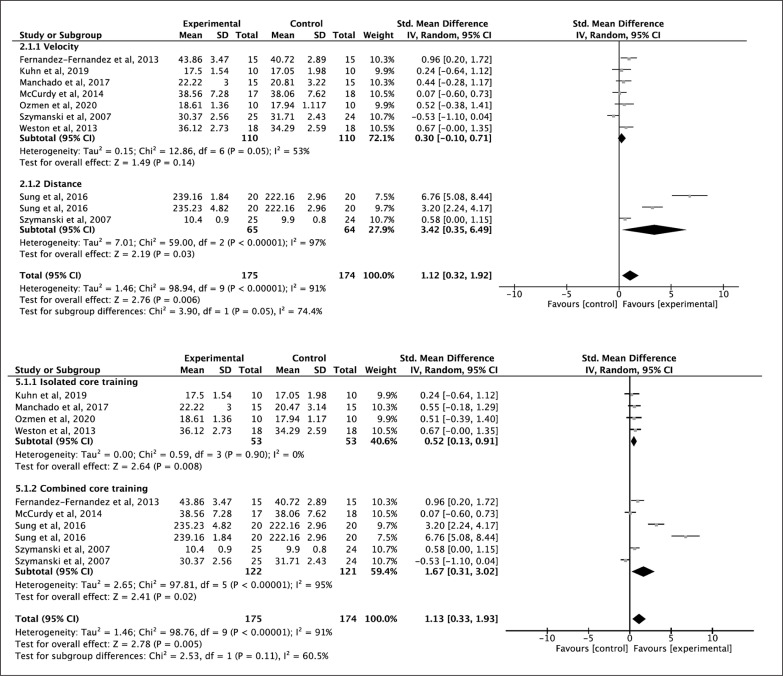
Forest plot of comparison of throw/hit velocity for core training vs control group and forest plot of comparison of throw/hit distance for core training vs control group (upper figure) and forest plot of comparison of isolated core training vs control group and forest plot of comparison of combined core training vs control group (down figure).

Throwing and hitting performance (velocity and distance outcomes) were combined into different subgroup analyses due to potential heterogeneity in results. Seven studies were included to analyze the effects of core training on throwing/hitting velocity with an SMD of 0.30 (p = 0.14; *I*^2^ = 53%) indicating a small but non-significant effect in favor of core training [30, 37, 39, 40, 42, 44, 49]. Two studies analyzed the effect of core on throwing/hitting distance indicating a large and significant effect in favor of core training with SMD of 3.42 points (p = 0.03; *I*^2^ = 97%) [15, 46]. All studies had a positive effect on the core training group except one study [49] (Figure 4 upper). The level of evidence according to the GRADE rating was low (Table 3).

When analyzed by the subgroup of ICT and CCT, four studies performed ICT with a SMD of 0.52 (p = 0.008; *I*^2^ = 0%) indicating a moderate and significant effect in favor of ICT on throwing/hitting performance. Four studies were included in the CCT subgroup with an SMD of 1.67 (p = 0.02; *I*^2^ = 95%) indicating a large and significant effect in favor of CCT for throwing/hitting performance (Figure 4 down).

In terms of intervention-related variables, large and significant effects were obtained with a frequency greater than two days per week (ES = 1.55), greater than 30 minutes (ES = 2.68), and a total volume greater than 16 weeks (ES = 1.55).

### Effects of Core Training Programs on Jumping Performance

**FIG. 5:**
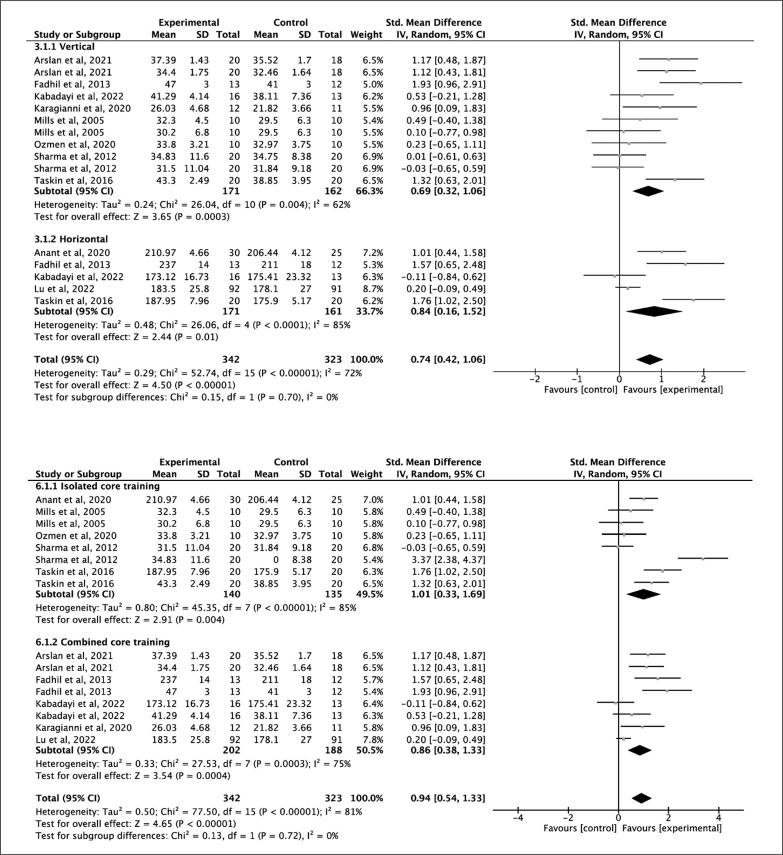
Forest plot of comparison of vertical jump for core training vs control group and forest plot of comparison of horizontal jump for core training vs control group and (upper figure) and forest plot of comparison of isolated core training vs control group and forest plot of comparison of combined core training vs control group (down figure).

The jumping performance was divided into two subgroups (vertical and horizontal jumping) for the analysis due to the heterogeneity of the results. Eight studies were analyzed for the vertical jump variable with an SMD of 0.69 points (p = 0.0003; *I*^2^ = 62%) indicating a moderate and significant effect in favor of core training with a [35, 36, 38, 40, 41, 47, 48, 51]. All studies included showed positive effects of core training on vertical jump except for one of the intervention groups in the study by Sharma et al., 2012. For the analysis of horizontal jumping 5 studies were assessed, with the SMD of 0.84 (p = 0.01; *I*^2^ = 85%) indicating a large and significant effect size in favor of core training [36, 41, 43, 51, 52] (Figure 5 upper). The level of evidence according to the GRADE rating was moderate and low quality of evidence, respectively (Table 3).

Furthermore, the effect of ICT and CCT on jumping performance was studied. A SMD of 1.01 (p = 0.004; *I*^2^ = 85%) indicates a large and significant effect in favor of ICT for jumping performance and an SMD of 0.86 (p < 0.001; *I*^2^ = 75%) indicating a large and significant effect size in favor of CCT for jumping performance (Figure 5 down). In terms of intervention-related variables a large and significant effect to equal to or less than 30 minutes per session (ES = 1.05) (Table 4).

## DISCUSSION

This meta-analysis aimed to synthesize as much scientific evidence as possible to determine the effect of core training on balance, throwing/hitting velocity, or distance and jumping in healthy subjects, identify the possible differences between isolated and combined core training on performance and study training, and sample variables related to performance. The main novelty of this meta-analysis was to analyze each performance variable separately. The current results show small to large effects, with significant differences in balance performance, throwing distance, and vertical and horizontal jump, and a small but non-significant effect on throwing velocity, in favor of core training. In addition, ICT and CCT with other types of training (jumps, upper or lower body training) have a moderate to large and significant effect on all performance variables analyzed.

Previous reviews and meta-analyses have focused on trunk muscle training and other performance variables like trunk and local muscle endurance, linear sprint speed and change of direction (COD) or agility, lower limb muscle power, maximal muscle strength, and sport-specific performance. However, when the sports performance variables have been evaluated, they have not been studied independently. Still, different performance variables from various sports such as swimming (time in the 50 m freestyle, time in the flight phase and recovering), handball (throwing velocity), golf (drive distance), athlete (running economy), and soccer (ball shooting speed) have been joined [10, 21]. Therefore, they concluded that the role of core training is more negligible in these variables; although, if these variables are analyzed separately, as has been done in this meta-analysis, core training improves performance.

Moreover, core strength training has been shown to have a small to medium effect on physical fitness and athletic performance measures in trained individuals [10]. In addition, age seems to be a variable that does not affect sports performance, except for COD and agility [21]. Similar results were found in our study, with no significant differences between youth and adults even though the effect sizes were larger for adults in throwing/hitting velocity or distance performance. Neither were significant differences in performance found according to gender. As Reed noted in his 2012 study, not all articles measure core strength or stability before and after intervention [9]. For example, only eleven of the 22 studies included in this review evaluated core strength or stability. In addition, of the studies that analyzed this, only two used the gold standard (isokinetic devices) [46, 49].

### Balance

Balance describes the dynamics of the body trying not to fall; therefore, it is crucial for daily life activities and sports [55]. The results of this meta-analysis show a large effect (ES = 1.17; p = 0.0001) in favor of core training groups. In the meta-analysis of Prieske et al., [10], the only balance test taken was the SEBT, and three studies were analyzed, although one of these studies did not perform the SEBT [38]. The results obtained do not agree with our studies, obtaining a small effect size in favor of core strength training. However, in our study, we added balance variables analyzed with the SEBT and the Y-balance test, and a large effect size was obtained in favor of core training.

Since the core muscles surround the center of mass in the lumbopelvic region [56], it should not be surprising that core training improves balance, i.e., improves the ability to keep the center of gravity stable within the base of support. The relationship between balance and injury risk has been previously established [57]. Indeed, the SEBT is considered a reliable measure and a valid dynamic test to predict the risk of lower extremity injury [58]. Recently, De la Motte et al. reported moderate evidence between poor balance test performance and increased risk of ankle injury [59]. Given the large effect of core training on balance, it is essential to consider the need to incorporate core training in sports in which balance plays a predominant role or has a higher incidence of lower extremity injury.

Furthermore, the results indicate that ICT and CCT have a moderate-large and significant effect on balance. Although there are no significant differences between the groups, the effect size of CCT (ES = 1.67) was larger than ICT (ES = 0.67). Therefore, greater improvements in balance will be obtained by training the core in combination with small-sized games, plyometrics, or lower limb training. The greatest effect of core training was found in combination with plyometrics, with an SMD of 2.72 points.

The methodology used to train the core was very varied, from 4 weeks to 8 weeks and with a frequency of 2 days a week to 5 days a week. It is essential to consider that none of the six studies included specifying the intensity at which the training was performed. Neither did they measure if core training produced differences in pre- and post-intervention strength, so we cannot be sure that the improvement in balance is due to core strengthening. Despite the varied training methodology, no significant differences were found for any of the variables related to the intervention. When observing the effect sizes, it could be concluded that improvement is greater with a frequency of 2 days a week, with a duration of more than 6 weeks, without the use of implements and a total volume equal to or less than 16 sessions, however, due to the high heterogeneity of the studies, it is not possible to obtain concluding data.

Future research should use implements that allow us to evaluate strength, such as functional dynamometers, and determine the training intensity, thus establishing whether the program corresponds to strength training [60, 61].

### Throwing/hitting velocity and distance

Results of our meta-analysis provide small effects in throwing/hitting velocity performance (ES = 0.30; p = 0.14); however, the results provide a large and significant effect of training core training on distance performance (ES = 3.42; p = 0.03). High heterogeneity was found in the data, and study quality was low. The high heterogeneity of the data could be because seven studies of 4 different sports (tennis, handball, baseball, and golf) were included in the study of throwing/hitting velocity performance. Only two studies were included in the distance performance, one measuring drive distance in golf and another medicine ball hitter’s throw in baseball players.

Throwing velocity or hitting velocity are determining variables in match performance [62], considered the most important in the offensive action in handball [63]. In tennis, the serve velocity is significantly related to winning the point [64, 65] and the position in the ranking [66]. In two studies, an isolated training core was found to have beneficial effects on throwing velocity [13, 24]. Similar results were found in our study, with a significant and moderate effect size in favor of isolated core training, although, when core exercise was combined, a large and significant effect size was obtained. However, Dahl et al. found no significant correlation between throwing velocity and core strength variables [22] and McCurdy et al., observed no significant effects of core intervention on tennis players’ performance [30]. The data obtained in our metanalysis cannot be compared with previous meta-analyses because they did not study athletic performance variables independently [10, 21].

The core training methodology used to improve throwing/hitting velocity performance was similar. In most studies, training time was 6 to 8 weeks with a frequency of 2/3 days a week. In the distance performance, since there are only two studies, data cannot be extracted from the training methodology since very few studies have analyzed these variables. However, both studies measured core strength before and after the intervention with the gold standard. In the statistical analysis of the variables related to training, better throwing/hitting velocity or distance performance is obtained when training sessions are carried out with a frequency greater than two days, a duration greater than 6 weeks and a session duration greater than 30 minutes.

### Jump

The jump ability is a requirement for success in several sports. This meta-analysis shows a moderate and significant effect on jumping performance with core training (ES 0.74; p < 0.00001). In addition, the relationship between trunk musculature and limb function has been extensively described [67, 68]; in particular, we have known that a trunk contraction is necessary prior to lower limb movement [69].

Previous reviews report a small and moderate effect of core training on vertical jumping [10, 21]. The data from this metanalysis show a moderate effect in favor of core training (ES 0.69; p = 0.0003). Butcher et al. reported an increase in take-off velocity in the vertical jump following nine weeks of core training [70]. In addition, there is a positive correlation between drop jump and isokinetic trunk extensor strength [71] and a strong correlation between isokinetic and isometric strength and CMJ [72]. Thus, core training could improve jumping by stabilizing the spine and pelvis, allowing a better transfer of force from the lower and upper extremities, and optimizing force production in activities such as jumping.

Concerning horizontal jump, the results of this meta-analysis show a large effect in favor of core training (ES = 0.84; p = 0.01). Performance in this test is 90% dependent on the flight distance, which is determined by the center of mass velocity at the take-off [73]. Furthermore, Takahashi et al. determined that the trunk muscles of long jumpers have a greater cross-sectional area than untrained individuals [74]. Besides, the size of the rectus abdominis and iliacus is associated with jumping performance. Thus, core training could be a strategy to increase horizontal jump performance. In addition, ICT had a larger effect size on vertical and horizontal jump performance than CCT despite not having a significant difference. As with the variable of throwing/hitting performance, the results obtained in this meta-analysis cannot be compared with previous meta-analyses because they analyze lower body muscle power, including power output, jumps, accelerations, and 3RM squat [10, 21].

The core training methodology used to improve vertical jump performance was very varied, from 6 weeks to 12 weeks and with a frequency of 2 days a week to 5 days a week. Only three studies assess core strength in pre-and-post-intervention [36, 40, 41]; however, none used the gold standard. In horizontal jump performance, two of the three included studies trained for eight weeks (three and five times a week) [40, 45], and one during 12 weeks with a frequency of 5 days a week [41]. In addition, two of the included studies measured core pre-and post-intervention. Anant et al. and Fadhil et al. found significant differences in lateral trunk strength and abdominal muscle endurance [41, 43]; however, none used the gold standard. In the statistical analysis of the variables related to training, the only significant variable is the duration of the session, obtaining improvements in sessions equal to or less than 30 minutes of core training.

### Limitations

This meta-analysis is not exempt from limitations, such as the use of only four databases, which may have limited the number of articles retrieved. In addition, the very different core training methodology may be a result of the high heterogeneity of studies in balance and vertical jump performance (*I*^2^ ≈ 60%) and distance (*I*^2^ = 97%). Publication bias was not performed in this analysis because it requires a minimum of 10 studies according to the Cochrane handbook [75]. Most studies included reported unclear randomization generation and allocation concealment. In articles with training to improve performance, it is challenging to blind the participants, so the risk of bias is high. It does not occur in other studies where a placebo can be used. However, we consider it a strength of this review to incorporate youth and adult populations from randomized studies. In contrast to other meta-analyses, this review analyzed specific performance variables and was not pooled as general categories.

## CONCLUSIONS

In conclusion, this systematic review and meta-analysis demonstrated that core training should be included in training sessions to improve athletic performance. Based on these findings, ICT and CCT have moderate and large effects on athletic performance, with no significant differences, although CCT performed better in balance and throwing/hitting and ICT in jumping performance. Therefore, for coaches of different sports in which one of these variables affects sports performance, training recommendations for balance improvement are inconclusive as there are no significant differences between the variables. However, if we consider the effect sizes, it is recommended to train 2 times a week with a duration of 6 weeks or less, without implements and a total volume equal to or less than 16 weeks. In the case of throwing/hitting performance, it is recommended to train more frequently than 2 days a week with sessions of more than 30 minutes and a total volume of more than 16 sessions and in jump performance, the recommendation would be to have sessions equal to or less than 30 minutes of core training.

No significant differences were found for age and gender for any of the performance variables analyzed.

## Figures and Tables

**TABLE S1:** Search strategy

Steps	Stategy	WOS	Scopus	Sports Discuss	PubMed	Total
1	“core muscle”	401	584	232	292	1509
2	“torso”	9594	15035	3458	202731	230818
3	trunk	76311	112484	8122	259555	456472
4	abdominal	321307	619166	8201	477254	1425928
5	S1 OR S2 OR S3 OR S4	402299	735331	17060	616890	1771580
6	“endurance training”	6013	10639	4051	5196	25899
7	stability	1881252	2226181	17615	782176	4907224
8	“resistance training”	12504	23293	10648	15262	61707
9	“strength training”	7994	8602	11199	5836	33631
10	“plyometric exercise”	261	1003	221	798	2283
11	S6 OR S7 OR S8 OR S9 OR S10	1904791	2262590	40414	804990	5012785
12	“athletic performance”	5497	18670	4996	15050	44213
13	performance	5292466	6275769	212943	3697887	15479065
14	“physical test”	1070	4937	136	201	6344
15	S12 OR S13 OR S14	5293188	6279313	213015	3697971	15483487
16	S5 AND S11 AND S14	1579	1481	605	3874	7539
	With filter After duplicates	772	944	587	502	2805 1929
